# Supplementary material for: A modular platform for one-step assembly of multi-component membrane systems by fusion of charged proteoliposomes
Source: Nat Commun. 2016 Oct 6;7:13025. doi: 10.1038/ncomms13025 (PMC5059690; doi:10.1038/ncomms13025)
Supplement: Supplementary Information — Supplementary Figures 1-9 [file ncomms13025-s1.pdf]

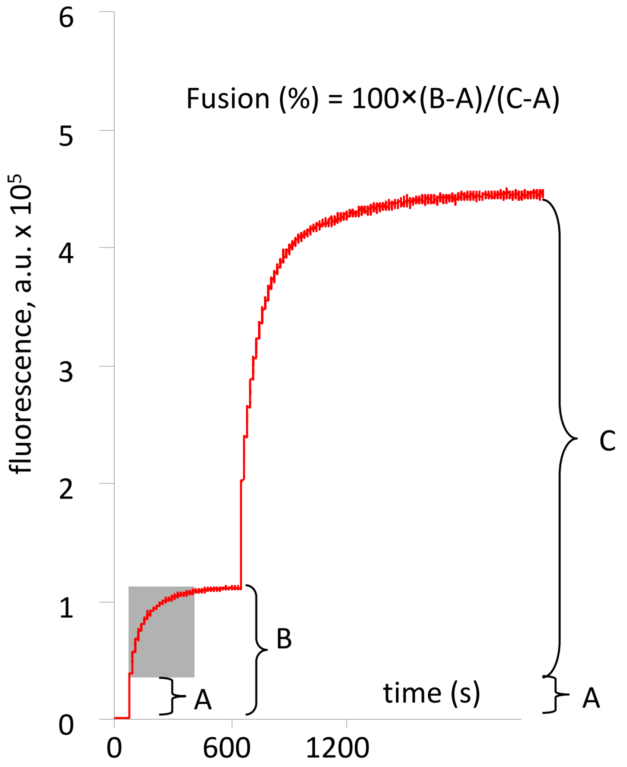

Supplementary Figure 1. Fusion of lipid vesicles studied with cobalt-calcein liquid content transfer assay. An example of fusion % calibration for the red trace shown in Fig. 1b. The difference between the maximal fusion signal B and the background signal A is divided by the equivalent maximal signal C following the release of encapsulated calcein by addition of EDTA/Triton X-100 and is multiplied by 100%. The trace from the shadowed region is shown in Fig. 1b.

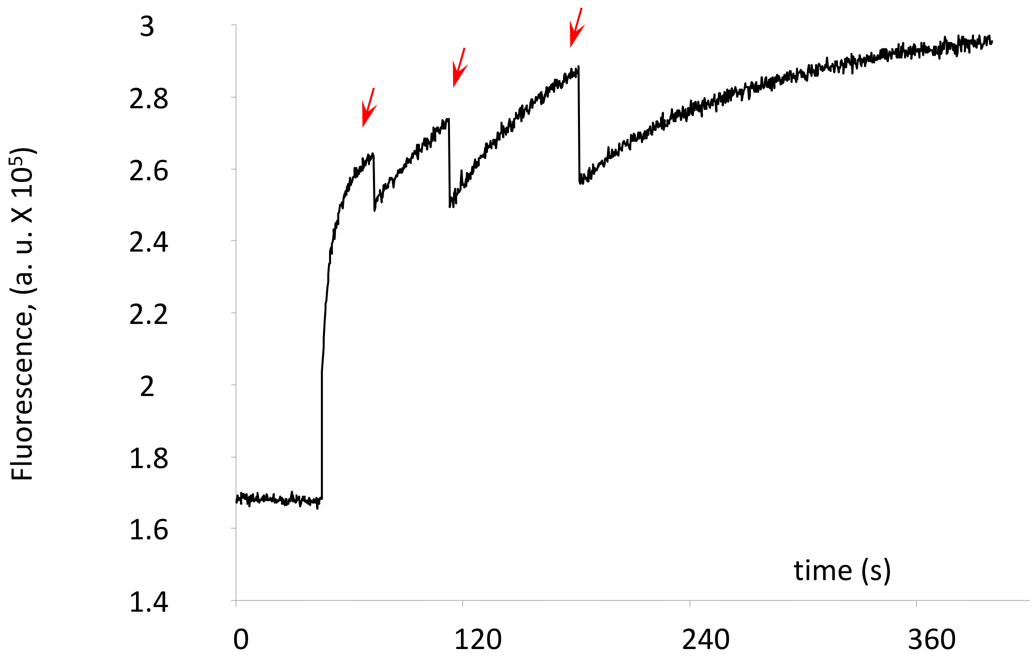

Supplementary Figure 2. Effect of external salt addition on vesicles fusion rate after initiation of vesicle fusion reaction studied with cobalt-calcein liquid content transfer assay. Addition of external 50 mM KCl three times (arrows) 30 seconds after complementary vesicles were mixed in 1 mM MOPS, pH 7.4 does not stop fusion.

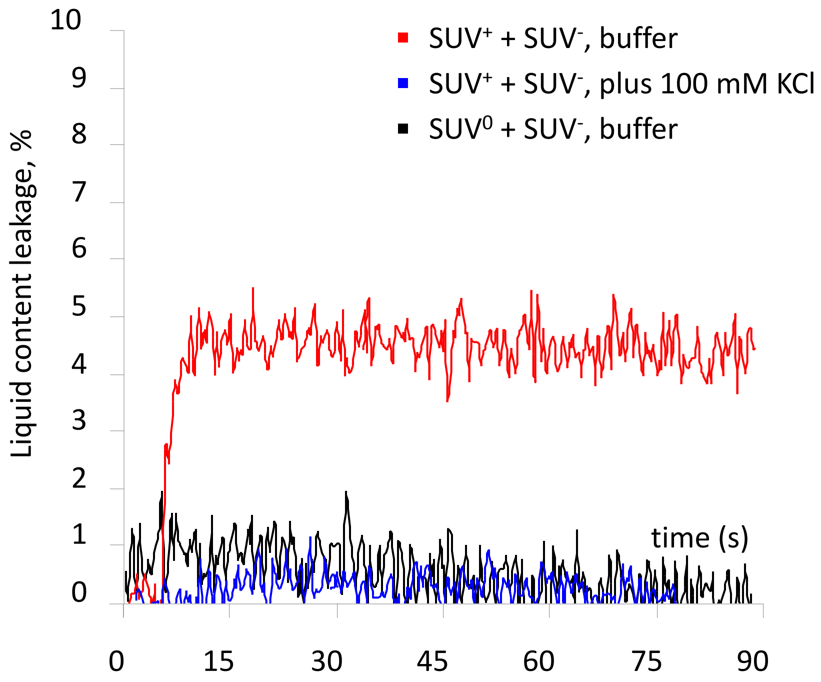

Supplementary Figure 3. Liquid content leakage during fusion of complementary SUV. 200 nm anionic SUV containing 100 mM calcein, 10 mM MOPS, pH 7.4 were fused with probe-free 200 nm cationic (red trace) or neutral (black) SUV in buffer (1 mM MOPS, pH 7.4), or probe-free cationic SUV in buffer supplemented with 100 mM KCl (blue trace)

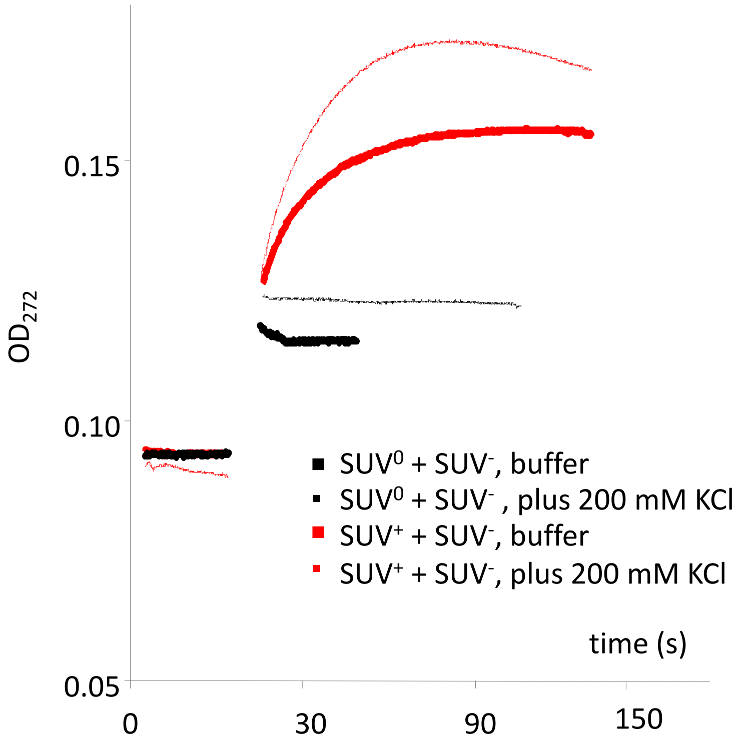

Supplementary Figure 4. Aggregation of complementary SUV. 200 nm anionic SUVs were mixed with 200 nm cationic (red curves) or neutral (black curves) SUV in buffer (1 mM MOPS, pH 7.4) (thick curves) or buffer plus 200 mM KCl (thin curves). The signal was followed by light absorption at 272 nm.

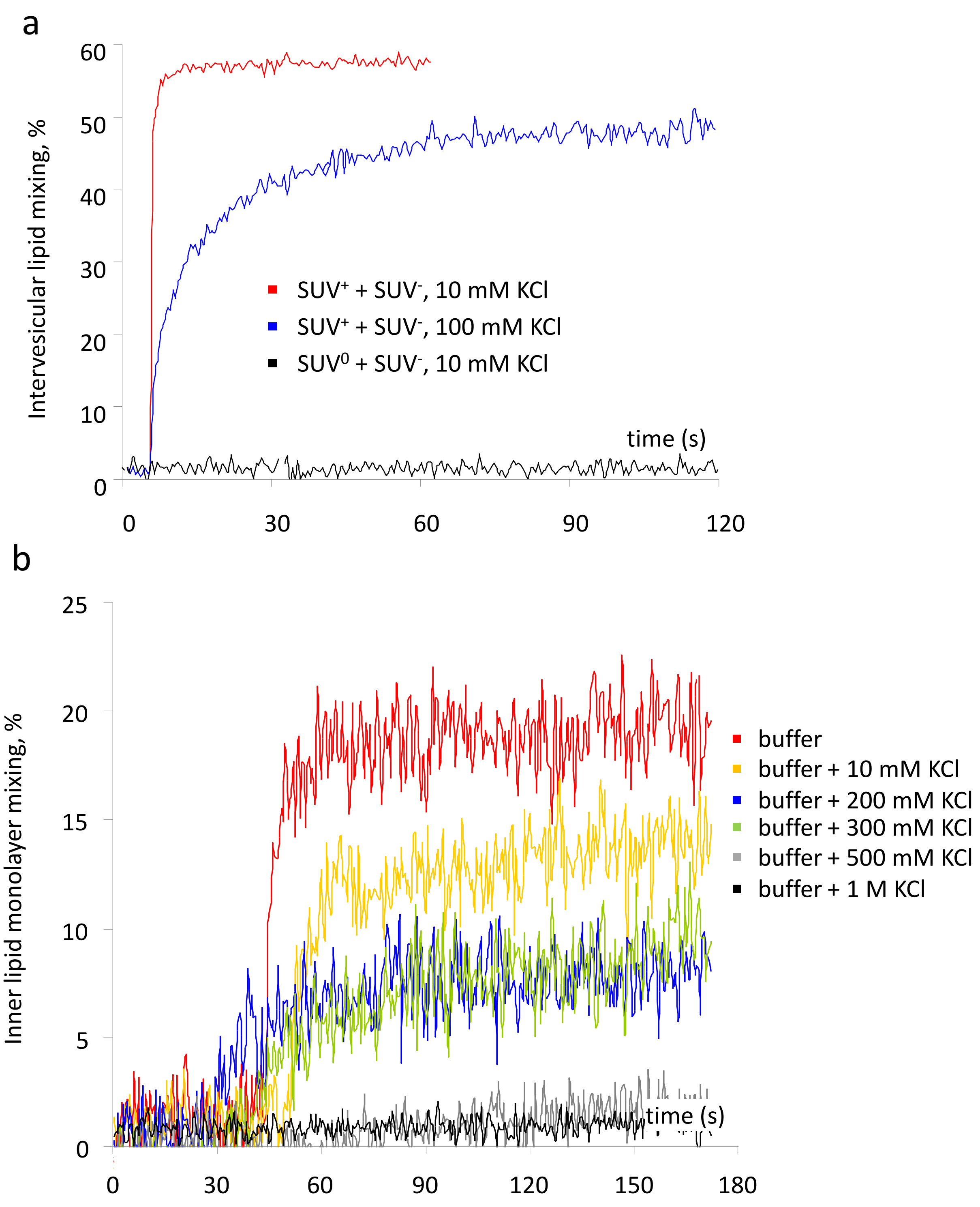

Supplementary Figure 5. Lipid mixing in complementary SUV. **(a) Intervesicular lipid mixing.** 200 nm anionic SUV containing 2% (weight fraction) NBD, 2% Rho-B, 25% POPA and 71% PC were mixed with 200 nm probe-free cationic (red trace) or neutral (black) SUV in buffer (1 mM MOPS, pH 7.4) containing 10 mM KCl, or with cationic SUV in buffer supplemented with 100 mM KCl (blue trace), at 1:4 ratio. **(b) Inner lipid monolayer mixing**. Same anionic SUV treated with sodium dithionite to quench fluorescence of their external lipid monolayer as described in Methods were mixed with probe-free 200 nm cationic SUV in buffer (1 mM MOPS, pH 7.4) containing various amounts of KCl, at 1:4 volume

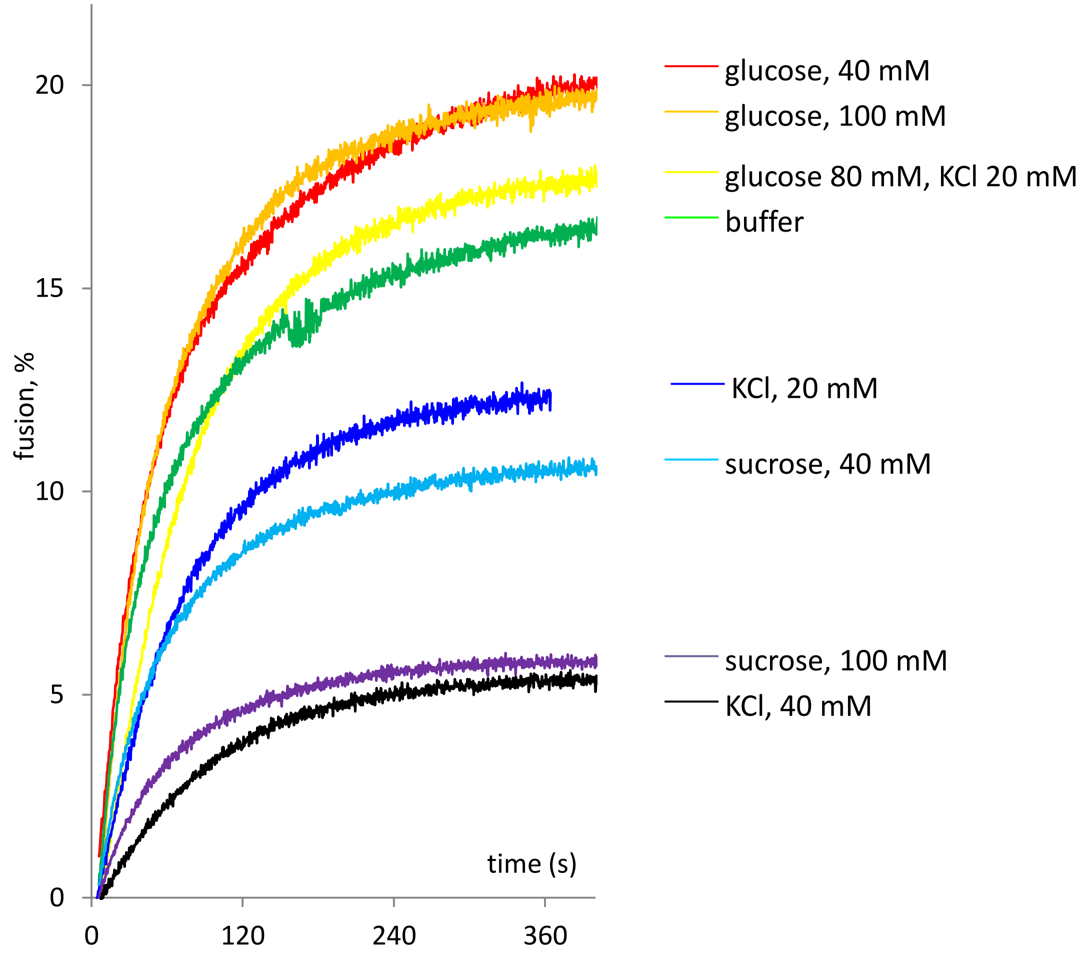

Supplementary Figure 6. Influence of osmolytes (glucose and sucrose) on vesicle fusion. 200 nm anionic SUV were fused with 200 nm cationic SUV in buffer (1 mM MOPS, pH 7.4) in presence of 40 or 100 mM glucose and sucrose as compared to fusion in 20 and 40 mM KCl.

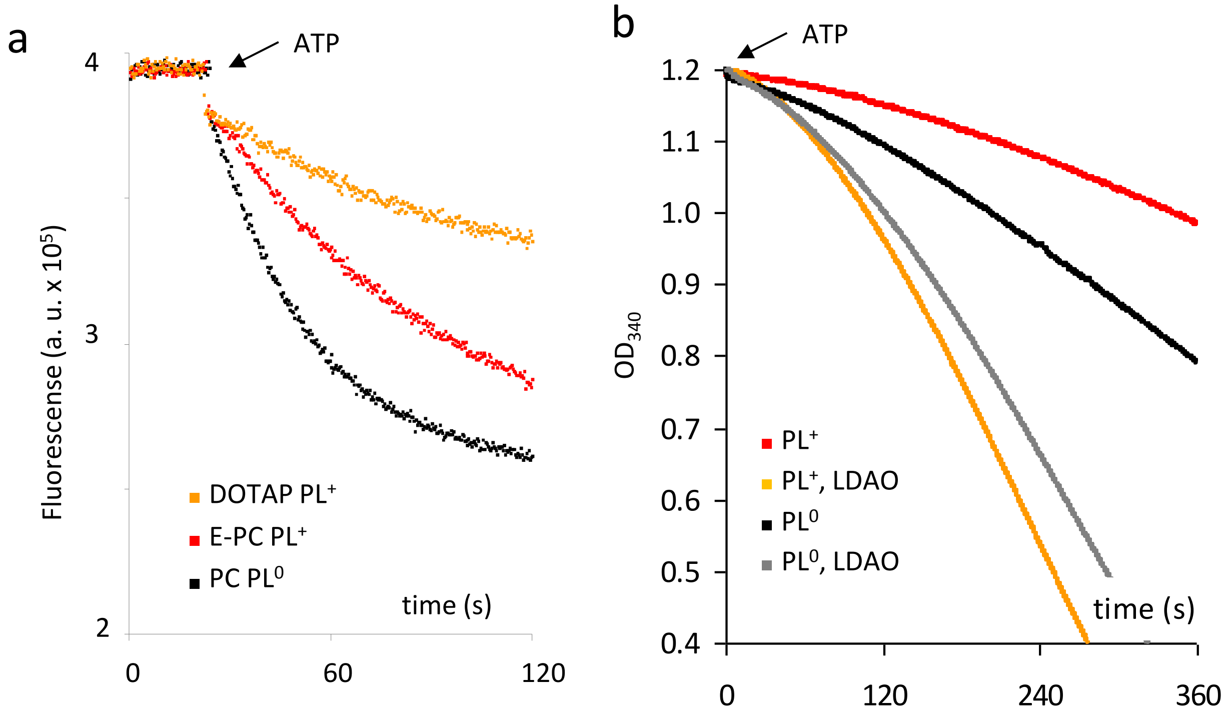

Supplementary Figure 7. Effect of bilayer charge on ATP hydrolase activity of F_1F_o ATP-synthase in neutral proteoliposomes (PL^0, PC only) or cationic proteoliposomes (PL^+, 50% PC: 50% cationic lipids DOTAP or E-PC, by weight). (a) ACMA quenching by DOTAP (orange trace), E-PC (red) and PC (black) PL. The proteoliposomes were formed by using the same amount of the protein and lipids as described in Methods, and the same volumes of proteoliposomes were used in the assay. Proton pumping was initiated by adding 0.2 mM ATP to the reaction medium (100 mM KCl, 1 mM $MgCl_2$, 50 mM MOPS pH 7.4). **(b)** ATP hydrolysis by PC^0 and E-PC PL^+ without (black, red) and with (grey, orange) 0.4% LDAO. The activity was measured with ATP regenerating system containing 100 mM KCl, 50 mM MOPS, pH 7.4, 2.5 mM $MgCl_2$, 1 mM ATP, 2 µM nigericin, 2 mM phosphoenolpyruvate, 0.2 mM NADH, 5 units/ml of pyruvate kinase and lactate dehydrogenase.

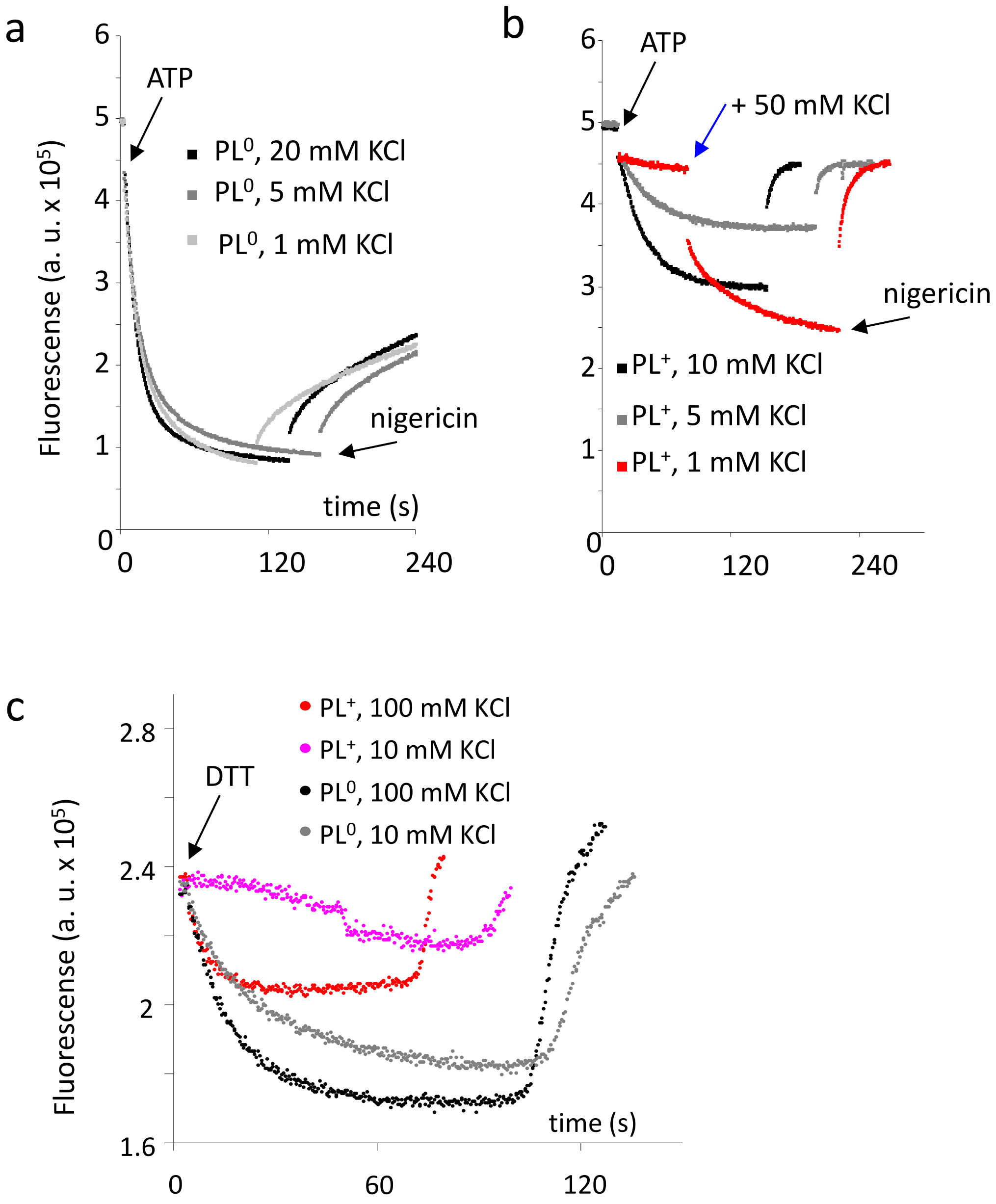

Supplementary Figure 8. Dependence of proton pumping by F_1F_o ATP-synthase (a, b) and bo_3-oxidase (c) in cationic (PL^+) and neutral (PL^0) proteoliposomes on KCl concentration. Proteoliposomes were assayed in 1 mM MOPS pH 7.4, 1 mM $MgCl_2$, and indicated concentrations of KCl. Proton gradient was dissipated by 2 µM nigericin. **(a)** ATP hydrolysis driven ACMA quenching by PL^0 is independent of the salt concentration. **(b)** ATP hydrolysis driven ACMA quenching by PL^+ is sensitive to low ionic strength and is minimal in 1 mM KCl (red trace); the pumping can be restored by addition of 50 mM KCl (blue arrow). **(c)** ACMA quenching driven by coenzyme-Q_1 oxidation and triggered by addition of DTT to bo_3-oxidase proteoliposomes. Proton pumping by PL^+ is weak (red) and low ionic strength sensitive (pink) in contrast to proton pumping by PL^0 (black, grey), which is not affected by low ionic strength.

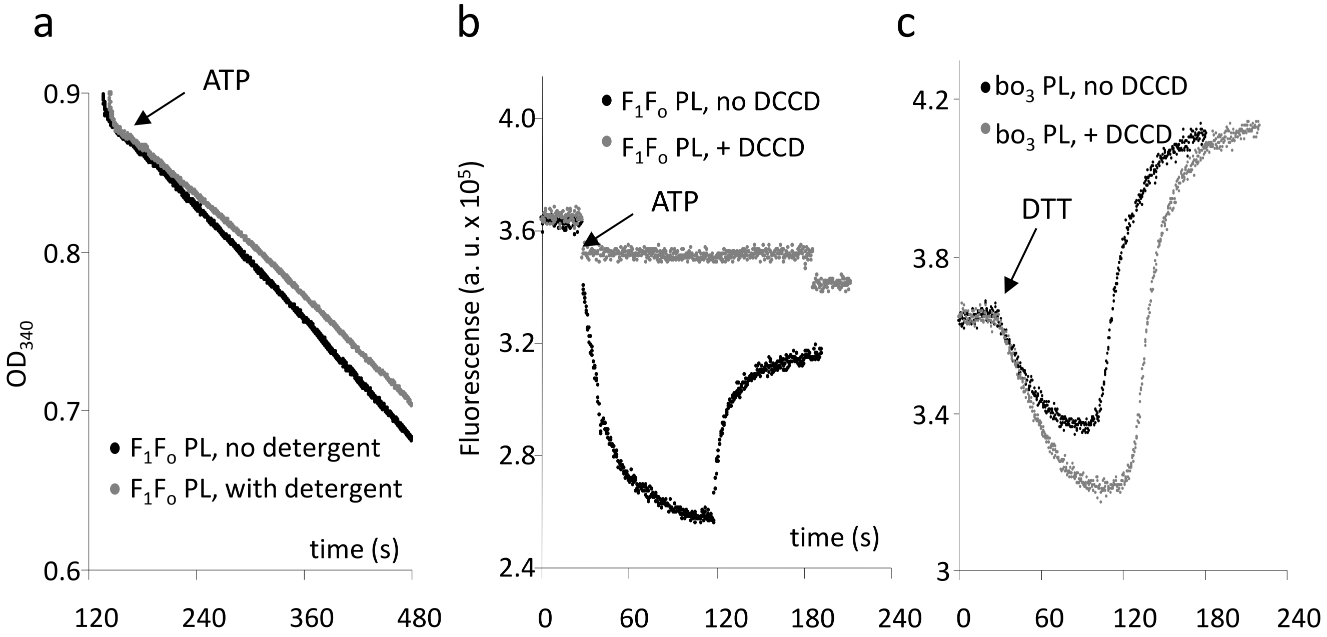

Supplementary Figure 9. Control experiments demonstrating unidirectional reconstitution of F_1F_o ATP-synthase into proteoliposomes (a), and DCCD-selective inhibition of proton pumping by F_1F_o (b) but not bo_3 oxidase (c). (a) ATP hydrolase activity of F_1F_o PL^0 with (grey) and without (black) vesicle disruption by the non-denaturing detergent 0.5% sodium cholate measured with ATP-regenerating system as described in Methods. (b) ATP hydrolysis driven ACMA quenching by F_1F_o ATP-synthase PL^0 (black) was completely abolished after 1 hour incubation with 50 µM DCCD (grey). (c) Coenzyme Q_1 driven ACMA quenching by bo_3 PL^0 is not sensitive to 50 µM DCCD .
